# *SGCD* Homozygous Nonsense Mutation (p.Arg97^∗^) Causing Limb-Girdle Muscular Dystrophy Type 2F (LGMD2F) in a Consanguineous Family, a Case Report

**DOI:** 10.3389/fgene.2018.00727

**Published:** 2019-01-23

**Authors:** Muhammad Younus, Farooq Ahmad, Erum Malik, Muhammad Bilal, Mehran Kausar, Safdar Abbas, Shabnam Shaheen, Mohib Ullah Kakar, Majid Alfadhel, Muhammad Umair

**Affiliations:** ^1^State Key Laboratory of Membrane Biology, Beijing Key Laboratory of Cardiometabolic Molecular Medicine, Institute of Molecular Medicine, Peking-Tsinghua Center for Life Sciences, PKU-IDG/McGovern Institute for Brain Research, Peking University, Beijing, China; ^2^Department of Biochemistry, Faculty of Biological Sciences, Quaid-i-Azam University, Islamabad, Pakistan; ^3^Department of Biochemistry, Shah Abdul Latif University Khairpur, Khairpur, Pakistan; ^4^DMLS Department, The University of Lahore, Islamabad Campus, Islamabad, Pakistan; ^5^Department of Higher Education, Government Girls Degree College Serai Naurang (Lakki Marwat), Peshawar, Pakistan; ^6^School of Life Sciences, Beijing Institute of Technology, Beijing, China; ^7^Division of Genetics, Department of Pediatrics, King Abdulaziz Medical City, Riyadh, Saudi Arabia; ^8^Medical Genomics Research Department, King Abdullah International Medical Research Center (KAIMRC), King Saud Bin Abdulaziz University for Health Sciences, Ministry of National Guard–Health Affairs (MNGHA), Riyadh, Saudi Arabia

**Keywords:** limb-girdle muscular dystrophies, LGMD, targeted NGS, *SGCD*, LGMD2F, homozygous variant, nonsense mutation

## Abstract

**Background:** Limb-girdle muscular dystrophy (LGMD) is an increasingly heterogeneous category of inherited muscle diseases, mainly affecting the muscles of shoulder areas and the hip, segregating in both autosomal recessive and dominant manner. To-date, thirty-one loci have been identified for LGMD including seven autosomal dominant (LGMD type 1) and twenty four autosomal recessive (LGMD type 2) inherited loci.

**Methodology/Laboratory Examination:** The present report describes a consanguineous family segregating LGMD2F in an autosomal recessive pattern. The affected individual is an 11-year-old boy having two brothers and a sister. Direct targeted next generation sequencing was performed for the single affected individual (VI-1) followed by Sanger sequencing.

**Results:** Targeted next generation sequencing revealed a novel homozygous nonsense mutation (c.289C>T; p.Arg97^∗^) in the exon 3 of the delta-sarcoglycan (*SGCD*) gene, that introduces a premature stop codon (TCA), resulting in a nonsense mediated decay or a truncated protein product.

**Discussion and Conclusion:** This is the first report of LGMD2F caused by an *SGCD* variant in a Pakistani population. The mutation identified in the present investigation extends the body of evidence implicating the gene *SGCD* in causing LGMD2F and might help in genetic counseling, which is more important to deliver the risk of carrier or affected in the future pregnancies.

## Introduction

Limb-girdle muscular dystrophies (LGMDs) are a group of an inherited muscle disorder, which is heterogeneous in nature, typically affecting the pelvic girdle (hips) and voluntary muscles of the shoulders. LGMDs primarily affects the proximal limb muscles however, other muscles like the respiratory and cardiac muscles also may get degenerated (Nigro and Savarese, 2014).

Limb-girdle muscular dystrophies can be broadly classified into two classes namely autosomal dominant form known as limb-girdle muscular dystrophies type 1 (LGMD1) and autosomal recessive form known as limb girdle muscular dystropies type 2 (LGMD2). LGMD1 can be further sub-classified into seven subtypes whereas LGMD2 into twenty four subtypes (Thompson and Straub, 2016).

Sarcoglycanopathies in humans are rare genetic disorders with an incidence of 1/178,000 live births (Fanin et al., 1997). Sarcoglycanopathies such as alpha (α), beta (β), gamma (γ), and delta (δ) sarcoglycans are a subset of recessive, severe form of LGMDs (LGMD2C-F) that present in early childhood (Magri et al., 2014). These encode single-pass transmembrane glycoproteins (α-, β-, δ-, γ-sarcoglycans), making up the tetrameric sarcoglycan-sarcospan complex (SGC). Sarcoglycans are basically N-glycosylated transmembrane proteins forming a hetero-tetrameric complex, which is mostly linked to the dystrophin–dystroglycan complex (Sandonà and Betto, 2009). SGC is critical for maintaining sarcolemmal stability and pathogenic sequence variants in the *SGCA*, *SGCB*, *SGCD*, or *SGCG* might result in non-assembly of the SGC (Shi et al., 2004; Tarakci and Berger, 2016).

The delta sarcoglycan (δ) gene (*SGCD*) is the largest LGMD gene spanning a genomic region of 433 kb on chromosome 5q33.3 and the fully functional transcript has a total of 9 exons. The second (2nd) intron of δ-sarcoglycan is one of the largest intron in human genome having a size of 164 kb. Both gamma (γ) and delta (δ) sarcoglycan can have a size of 35 kDa and are homologous to each other (Dickens et al., 2002).

Here, we characterized a consanguineous Pakistani family with LGMD2F caused by a novel homozygous nonsense mutation (c.289C>T; p.Arg97^∗^) in the exon 3 of the *SGCD* gene (NM_001128209.1).

## Case Presentation

**FIGURE 1:**
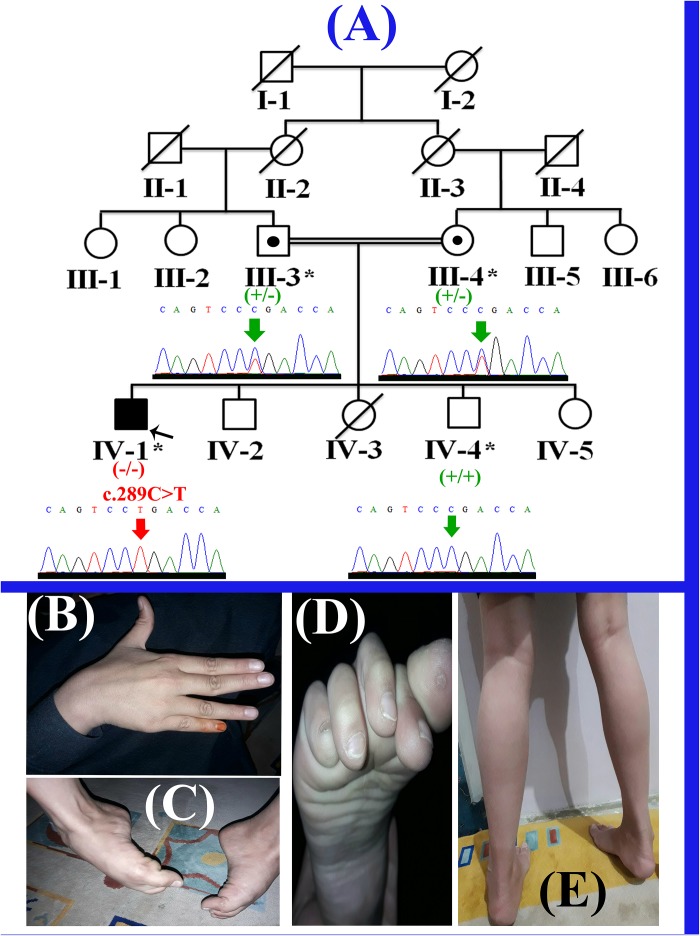
**(A)** Pedigree of the present family, segregating autosomal recessive form of LGMD2F. Clear circles and squares represent normal females and males, respectively. Black symbol represents the affected individual (proband). Double lines indicate consanguineous union. Individual numbers labeled with asterisks, indicate those who were available for the present study. **(B)** Affected individual’s (IV-1) hand showing contractures. **(C,D)** Right foot of the affected individual (IV-1) showing fixed flection deformity and stiffness of the toes. **(E)** Feet showing the weakness in the gastrocnemius muscle and the inability to stand without support.

The present study describes a single affected boy (IV-1; 11 years) that belongs to a consanguineous Pakistani family (Figure 1A). The affected individual (IV-1) has three normal siblings (two boys (IV-2; 10 years; IV-4; 7 years) and a girl (IV-5; 1.5 years). The proband (IV-1) presented here, was the first son of consanguineous Pakistani parents and had a birth weight of 3.5 kg (50th centile). His first symptoms presented at 3–4 years of age when his parents noted different walking style and less weight gain. He started sitting without assistance at 8 months and walking at 15 months of age. He started to run at 2.5 years of age, never jumped or hopped and suffered frequent 2–3 falls per day. The affected boy had difficulty in walking and climbing stairs since 3 years of age. He has relatively normal extensor muscles of the fingers, wrist, toes flexors and hip abductors, with severe weakness of other muscles (Figures 1B–E). The cardiac review suggested mild cardiac hypertrophy, muscular weakness, normal peripheral pulse and no murmurs were observed. Venus pressure was normal and signs of heart failure were not observed. He was diagnosed having LGMD on the basis of the observed phenotypes, and molecular diagnosis. Detailed clinical findings observed in the proband (IV-1) have been described in the Supplementary Table 1.

## Echocardiography

Echocardiography showed situs solitus, normal atrial and ventricular arrangements. The cardiac chambers were not enlarged. The inferior vena cava (IVC) was not dilated and there was a pulsatile descending aorta. Views of the atrial septum were limited and there was suspicion of a tiny patent foramen ovale (PFO) with a left to right shunting. The mitral valve was normal with no significant regurgitation or stenosis. The tricuspid valve was also normal with no signs of stenosis or regurgitation and no ventricular septal defect was observed. The aortic valve and the pulmonary valve were normal with only trivial pulmonary regurgitation and the left ventricle functioned normally. The mitral valve inflow Doppler demonstrated a wave of 0.47 m/s and an E wave of 0.67 m/s. The E/A ratio was 1.42 and the mitral valve acceleration time was 164 ms. Tissue Doppler indices of the left ventricular free wall demonstrated a normal pattern of E/A, and S waves, while the S wave velocity was around 10 cm/sec. The right ventricular function was normal with a Tricuspid annular plane systolic excursion (TAPSE) of 15.5 mm, while the Isovolumic relaxation time (IVRT) of the left ventricle was 83 ms and E/e prime ratios did not suggest that left atrial pressures were significantly elevated. Left ventricular systolic function was normal. The left ventricular end diastolic diameter was observed 24 mm having fractional shortening of 33%. In the short axis view prolapse of the mitral valve was observed, not causing any significant regurgitation. The coronary arteries were normal. There were confluent pulmonary arteries with no patent ductus arteriosus, normal left sided arch and no pericardial effusion. Furthermore, ECG which did not reveal any significant signs of arrhythmia or ischaemia. The affected individual (IV-1) muscle weakness and lack of mobility limit his physical capabilities but he has not experienced any significant signs of exercise incompetence, although he suffered from episodes of syncope. A muscle biopsy of the affected individual was not available. Parents of the affected individual were healthy and showed no abnormality.

## DNA Extraction and Targeted Next Generation Sequencing

The present study was performed following the declaration of Helsinki protocols, and written informed consent for conducting the study and for publication of photographs was obtained from the parents. Peripheral blood samples were obtained from four individuals including three unaffected (III-3, III-4, IV-4) and single affected (IV-1) individual in an EDTA containing Vacutainer tubes (BD, Franklin Lakes, NJ, United States). Extraction of genomic DNA was performed using GenElute^TM^ Blood Genomic DNA Kit (Sigma-Aldrich, MO, United States) and quantified using Nanodrop-1000 spectrophotometer (Thermal Scientific, Wilmington, MA, United States).

**FIGURE 2:**
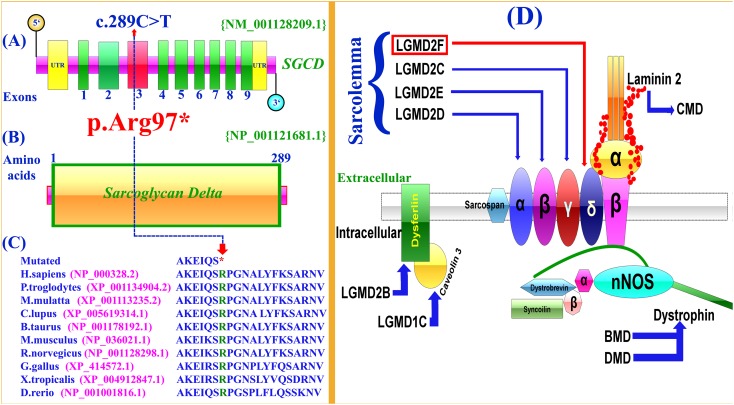
Schematic representation of exons and protein product of *SGCD* gene. **(A,B)** Arrows show the position the mutation (c.289C>T; p.Arg97^∗^) identified in the present study. **(C)** Conservation of the Arg (R) 97 amino acid across different species. Notably, the nonsense mutation (c.298C>T) results in a premature stop codon (TCA). **(D)** Schematic representation of the sarcoglycan complex. The intracellular and extracellular regions of sarcoglycans are separated by a cell membrane. Different sarcoglycans, α, β, γ, δ, dysferlin, caveolin 3, dystrophin and laminin are shown and associated disorders are also shown using arrows.

Selective analysis of 31 genes associated with LGMD was performed using SureSelect focused Exome +1 capture (Agilent Technologies, CA, United States). Sequencing was performed using NextSeq 500 platform (Illumina, San Diego, CA, United States) using Burrows-Wheeler Aligner (BWA v 0.7.5), and all reads were aligned against human assembly hg19 (GRCh37). Variant calling (Free bayes), variant annotation (Alamut-batch) was performed using in-house pipeline, with a minimum sensitivity of 99.9%. Since the pedigree clearly depicted an autosomal recessive inheritance of the phenotype (Figure 1A), therefore to identify a putative pathogenic homozygous recessive variants, we first searched for the homozygous variants in the 24 known LGMD genes (Supplementary Table 2). To search for disease causing variants, targeted NGS and screening was performed as described previously (Umair et al., 2017a). Variants screening was performed based on autosomal recessive inheritance pattern for the LGMD phenotype, rare variants and OMIM list of LGMD genes. We focused only on pathogenic, disease causing non-synonymous (NS) variants, causing nonsense, missense, splice acceptor/donor site, short frame-shift coding deletions (indel) or insertions and large deletions/duplications. Considering LGMD as a rare disorder, it is highly unlikely that the disease causing variant could be present in homozygous form in general population, therefore the data was cross-matched with online public databases. Following step-by-step filtering process for screening homozygous and compound heterozygous variants in the known genes causing LGMDs, a nonsense mutation (c.289C>T; p.Arg97^∗^) was identified in the exon 3 of the *SGCD* gene, located on chromosome 5q33.2-q33.3 (Figures 2A,B).

## *In Silico* Analysis

The variant (c.289C>T) was not found in homozygous state in the dbSNP^1^, 1000 Genomes^2^, EVS^3^, genomAD^4^, and Exome Aggregation Consortium ExAC^5^. In the ExAC database containing more than 60,000 human exome data, the identified *SGCD* variant (c.289C>T) was found in heterozygous state with an allelic frequency of 0.000153 in European (Finnish) population.

The identified variant was predicted to be deleterious using different online available bioinformatics tools such as Mutation Taster^6^, Sorting Intolerant From Tolerant (SIFT)^7^, and Polymorphism Phenotyping V2 (PolyPhen-2)^8^. The variant is also highly conserved across multiple species (Figure 2C). To exclude polymorphic nature of the variant, it was cross checked within different online variant databases, 160 in-house exome (Pakistan) from unrelated Pakistani individuals, and 185 ethnically matched controls.

## Sanger Sequencing Validation

The homozygous sequence variant (c.289C>T; p.Arg97^∗^), identified in the present study was verified using Sanger sequencing in all the available family members and the variant perfectly segregated with the disease phenotype (Figure 1A). Genomic sequence of the *SGCD* gene was retrieved from the University of California Santa Cruz (UCSC) genome database browser^9^. Primer sequences for PCR amplification of the exon 3 were designed using Primer3 software^10^. Primers sequences used for the present study include (Forward primer-F1: GGAATGGGAAACCTGAGG; Reverse primer-R1: AGCTGGGTAGCAGGGGTTG). Sanger sequencing procedure has been described earlier (Umair et al., 2016). Mutation was identified via BIOEDIT sequence alignment editor version 6.0.7 (Ibis Biosciences Inc., Carlsbad, CA, United States).

## Discussion

In the present study, we performed genetic and molecular analysis on a consanguineous family having a single affected individual (IV-1), segregating LGMD2F in an autosomal recessive manner. Consanguineous marriages are preferred in a county like Pakistan due to its socioeconomic and cultural obligations (Umair et al., 2018). The coefficient of inbreeding is increased by consanguinity, which increases the presence of variant in a homozygous state. Pakistani population displays rare genetic ailments due to higher coefficient of inbreeding. In such a situation, WES is an efficient, fast and economical strategy for detection of pathogenic disease-causing variants (Umair et al., 2017b). LGMD phenotypes observed in the affected individual were similar to those reported previously (Dinçer et al., 2000). Cardiomyopathy is not a core component of sarcoglycanopathies, still there have been reports of several patients without cardiac involvement and mutations in the *SGCD* gene have also been reported to cause autosomal-dominant dilated cardiomyopathy (DCM) independently from LGMD phenotypes (Tsubata et al., 2000).

LGMD2F is the fourth sarcoglycanopathy (LGMD2C to LGMD2F) type, characterized when the causative mutation in the δ-SG gene was identified for the first time in Brazilian LGMD patients with a Duchenne muscular dystrophy (DMD) type features linked to chromosome 5q33-34. Since then, a large number of mutations causing both LGMD2F and δ-SG-associated cardiomyopathy have been described (Leiden Muscular Dystrophy database^11^).

In the present investigation, targeted next-generation sequencing revealed a novel homozygous nonsense mutation (c.289C>T; p.Arg97^∗^), segregating with the disease phenotype within the family. To-date only twelve mutations have been identified in the *SGCD* gene associated with LGMD including four nonsense, five missense, two small deletions and one gross deletion (Supplementary Table 3). *In silico* analysis, revealed that the variant (c.289C>T; p.Arg97^∗^) might result in truncated SGCD protein lacking 192 conserved amino acids and most likely degraded by nonsense mediated mRNA decay (NMD).

Six sarcoglycans, α, β, γ, δ, η and ζ have been identified so far. Mutations in the genes for α, β, γ, and δ-sarcoglycan causes heterogeneous group of autosomal recessive limb-girdle muscular dystrophies (LGMD2C-F) (Bönnemann et al., 1995; Noguchi et al., 1995). The sarcoglycan deficient LGMDs are characterized by progressive weakness of the pelvic and shoulder girdle musculature with symptoms ranging from mild to highly severe (Zatz et al., 2003). Patients with LGMD2C-F, in particular with β-sarcoglycan deficient (LGMD2E) and δ-sarcoglycan-deficient (LGMD2F), often develop a progressive and potentially fatal DCM in association with muscular dystrophy (Politano et al., 2001).

The sarcoglycans are part of the dystrophin-glycoprotein complex (DGC), an oligomeric complex spanning the plasma membrane of skeletal and cardiac muscle fibers. Sarcoglycans plays a key role in the stability of cytoskeleton of muscle membrane. In the plasma membrane of muscle, they form a tetrameric complex that stabilizes the association of dystrophin with α and β dystroglycans (Figure 2D).

*SGCD* mutations causing LGMD2F and its clinical presentation are largely similar among the four sarcoglycanopathies (Nigro and Savarese, 2014). It is assumed that integrity of the sarcoglycan complex is crucial for maintaining the mechanical and non-mechanical linkage between the subsarcolemmal cytoskeleton and the extracellular matrix (Ozawa et al., 2005; Heydemann and McNally, 2007). *Sgcd* knockout mice also showed both muscular dystrophy (Loss of vascular smooth muscle SG-SSPN complex) and cardiomyopathy (focal areas of necrosis as the histological hallmark in cardiac and skeletal muscle) features (Coral-Vazquez et al., 1999).

In conclusion, we have reported a consanguineous Pakistani family having a novel *SGCD* variant causing LGMD2F. It is evident that δ-SG pathogenesis is complex and multifactorial which might require combinatorial therapeutic strategies. The clinical and molecular manifestation of such a heterogeneous disease is required timely, that might also involve different genetic modifiers. Still, the primary cure through gene or cell therapy may be beneficial for the patient in the future. This molecular finding increases the mutational spectrum of *SGCD* related pathogenesis and also provides proper genetic counseling to the affected family.

## Author Contributions

MY and MU drafted the manuscript. MY, FA, MB, SA, MK, SS, and EM collected the samples and clinical data, analyzed the data, and performed the experiments. SA, EM, and MUK analyzed the genomic data. MF, MA edited the manuscript. MU and MA conceived and designed the work.

## Conflict of Interest Statement

The authors declare that the research was conducted in the absence of any commercial or financial relationships that could be construed as a potential conflict of interest.

## Footnotes

1 http://www.ncbi.nlm.nih.gov/SNP/

2 http://www.1000genomes.org

3 http://evs.gs.washington.edu/EVS/

4 http://gnomad.broadinstitute.org/

5 http://exac.broadinstitute.org/

6 http://www.mutationtaster.org/

7 https://sift.bii.a-star.edu.sg/

8 http://genetics.bwh.harvard.edu/pph2/

9 http://genome.ucsc.edu/cgi-bin/hgGateway

10 http://bioinfo.ut.ee/primer3-0.4.0/

11 http://www.dmd.nl/
