# Supplementary material for: SGCD Homozygous Nonsense Mutation (p.Arg97∗) Causing Limb-Girdle Muscular Dystrophy Type 2F (LGMD2F) in a Consanguineous Family, a Case Report
Source: Front Genet. 2019 Jan 23;9:727. doi: 10.3389/fgene.2018.00727 (PMC6354032; doi:10.3389/fgene.2018.00727)
Supplement: Supplementary file 1 [file Table_1.docx]

**Supplementary Table 1:** Main clinical findings observed in the proband IV-1.

| **Features observed** | **Present study** |
| --- | --- |
| **Consanguineous pedigree** | **Yes** |
| **Mutation type** | **Nonsense** |
| **Variant** | **c.289C>T;** |
| **Protein change** | **p.Arg97*** |
| **Race** | **Pakistani** |
| **Sex (Male: Female)** | **male** |
| **Age** | **11.5** |
| **Weight** | **27.7kg (9^th^-25^th^ centile)** |
| **Height** | **133.4cm (9^th^-25^th^ centile)** |
| **Head circumference** | **50.5cm (2^nd^ centile)** |
| **Wheelchair bound** | **No** |
| **Scoliosis** | **Yes** |
| **Shoulder girdle weakness** | **Yes** |
| **Dentinogenesis imperfecta** | **No** |
| **Pelvic girdle weakness** | **yes** |
| **Hypermobility of joints** | **No** |
| **Craniofacial features affected** | **No** |
| **Hearing impairment** | **No (Normal)** |
| **Growth retardation** | **No (Normal height)** |
| **Intellectual development** | **Normal** |
| **Cardiac impairment** | **No** |
| **Ocular findings** | **Normal** |
| **Thyroid stimulating hormone (THS)** | **2.3mU/L (Normal;<0.6mU/L)** |
| **Serum CK** | **18SU (20SU for children; 10SU for Adults)** |
| **Serum VZV IgG** | **286mlU/ml** (**Normal: >150mlU/ml)** |
| **IGF-1 (ng/μl)** | **186 ng/μl (Normal; IGF-1: males, 14 years (102–520 ng/μl)** |
| **PRL (ng/dl)** | **202 ng/dl (PRL: males (42.5–414 ng/dl)** |
| **Vitamin D** | **47nmol/L (Normal; 25-50 nmol/L)** |
| **Free T4 test** | **17.0 pmol/L (Normal;10.8-19.0 pmol/L)** |
