# Supplementary material for: SGCD Homozygous Nonsense Mutation (p.Arg97∗) Causing Limb-Girdle Muscular Dystrophy Type 2F (LGMD2F) in a Consanguineous Family, a Case Report
Source: Front Genet. 2019 Jan 23;9:727. doi: 10.3389/fgene.2018.00727 (PMC6354032; doi:10.3389/fgene.2018.00727)
Supplement: Supplementary file 2 [file Table_2.docx]

**Supplementary Table 2: Autosomal dominant (AD) and autosomal recessive (AR) LGMDs**

| **S.No.** | **LGMD (OMIM #)** | **Inheritance** | **Key clinical features** | **Locus (gene)** | **Protein** |
| --- | --- | --- | --- | --- | --- |
| **1** | LGMD1A (159000) | AD | Proximal and distal weakness, cardiomyopathy, neuropathy, dysarthria and facial weakness | 5q 22–34 (*TTID*) | Myotilin |
| **2** | LGMD1B (159001) | AD | Broad clinical spectrum, scapuloperoneal and humeral weakness, joint contractures, rigid spine, dilated cardiomyopathy, arrhythmias with atrioventricular block, atrial fibrillation, sudden cardiac death, lipodystrophy, neuropathy | 1q 11–21 (*LMNA*) | Lamin A/C |
| **3** | LGMD1C (607801) | AD | Proximal and distal muscle weakness, myalgia, rippling muscle disease, percussion-induced rapid contractions | 3p25 (*CAV3*) | Caveolin 3 |
| **4** | LGMD1D (603511) | AD | Proximal and distal weakness, in some patients dysphagia and cramps | 7q (*DNAJB6*) | HSP40 |
| **5** | LGMD1E (601419) | AD | Broad phenotypic spectrum, proximal and distal weakness, cardiomyopathy, arrhythmia, respiratory involvement | 6q23  (*DES*) | Desmin |
| **6** | LGMD1F (608423) | AD | Proximal>distal weakness, contractures | 7q32 (*TNPO3*) | Transportin 3 |
| **7** | LGMD1G (609115) | AD | Proximal>distal weakness, cataracts | 4q21 (*HNRNPDL*) | Heterogeneous nuclear ribonucleoprotein D‑like protein |
| **8** | LGMD2A (253600) | AR | Proximal weakness, contractures | 15q15–21 (*CAPN3*) | Calpain 3 |
| **9** | LGMD2B (253601) | AR | Proximal and distal weakness, asymmetry | 2p13 (*DYSF*) | Dysferlin |
| **10** | LGMD2C (253700) | AR | Proximal weakness, respiratory and cardiac involvement | 13q12 (*SGCG*) | γ‑Sarcoglycan |
| **11** | LGMD2D (608099) | AR | Proximal weakness, respiratory involvement | 17q 12–21 (*SGCA*) | α‑Sarcoglycan |
| **12** | LGMD2E (604286) | AR | Proximal weakness, respiratory and cardiac involvement | 4q12 (*SGCB*) | β‑Sarcoglycan |
| **13** | LGMD2F (601287) | AR | Proximal weakness, respiratory and cardiac involvement | 5q33–34 (*SGCD*) | δ‑Sarcoglycan |
| **14** | LGMD2G (601954) | AR | Proximal weakness, cardiac involvement | 17q11–12 (*TCAP*) | Telethonin |
| **15** | LGMD2H (254110) | AR | Proximal weakness | 9q31–34 (*TRIM32*) | Tripartite motif containing 32 |
| **16** | LGMD2I (606596) | AR | Proximal weakness, calf hypertrophy, respiratory involvement, cardiomyopathy, myalgia | 19q13 (*FKRP*) | Fukutin-related protein |
| **17** | LGMD2J (608807) | AR | Proximal>distal weakness | 2q (*TTN*) | Titin |
| **18** | LGMD2K (609308) | AR | Proximal weakness, mental retardation | 9q34 (*POMT1*) | Protein-*O*‑mannosyl transferasel |
| **19** | LGMD2L (611307) | AR | Proximal>distal weakness, lower limbs>upper limbs, adult onset | 11p12–13 (*ANO5*) | Anoctamin 5 |
| **20** | LGMD2M (611588) | AR | Axial and proximal>distal weakness, low IQ, cardiomyopathy | 9q31 (*FKTN*) | Fukutin |
| **21** | LGMD2N (613158) | AR | Proximal weakness, calf hypertrophy | 14q24 (*POMT2*) | Protein-*O*‑mannosyl transferase 2 |
| **22** | LGMD2O (613157) | AR | Proximal weakness, calf hypertrophy | 1p34 (*POMGnT1*) | Protein-*O*‑linked mannose β1,2 *N*‑acetylglucosaminyl transferase |
| **23** | LGMD2P (613818) | AR | Proximal>distal weakness, mental retardation, small head size | 3p21 (*DAG1*) | Dystroglycan |
| **24** | LGMD2Q (613723) | AR | Proximal>distal weakness | 8q24 (*PLEC1*) | Plectin |
| **25** | LGMD2R (615325) | AR | Proximal>distal weakness, atrioventricular conduction block, cardiomyopathy, facial involvement, respiratory involvement | 2q35 (*DES*) | Desmin |
| **26** | LGMD2S (615356) | AR | Proximal weakness, CNS involvement | 4q35 (*TRAPPC11*) | Transport protein particle complex 11 |
| **27** | LGMD2T (615352) | AR | Proximal weakness, calf hypertrophy, learning difficulties, cardiomyopathy | 3p21 (*GMPPB*) | GDP-mannose pyrophosphorylase B |
| **28** | LGMD2U (616052) | AR | Proximal>distal weakness, cardiac involvement | 7p21 (*ISPD*) | Isoprenoid synthase domain |
| **29** | LGMD2V | AR | Proximal and axial>distal weakness | 17q25 (*GAA*) | α-1,4 glucosidase |
| **30** | LGMD2W (616827) | AR | Proximal weakness, cardiomyopathy and triangular tongues | 2q14 (*LIMS2*) | Lim and senescent cell antigen-like domains 2 |
| **31** | LGMD2X (616812) | AR | Proximal weakness, arrhythmias with atrioventricular block | 6q21 *(BVES)* | Blood vessel endocardial substance |
