# Supplementary material for: SGCD Homozygous Nonsense Mutation (p.Arg97∗) Causing Limb-Girdle Muscular Dystrophy Type 2F (LGMD2F) in a Consanguineous Family, a Case Report
Source: Front Genet. 2019 Jan 23;9:727. doi: 10.3389/fgene.2018.00727 (PMC6354032; doi:10.3389/fgene.2018.00727)
Supplement: Supplementary file 3 [file Table_3.docx]

**Supplementary Table 3: Mutations to date in the *SGCD* gene causing LGMD.**

| **S.No.** | **Mutation type** | **Nucleotide change** | **Protein change** | **Reported phenotype** |
| --- | --- | --- | --- | --- |
| 1 | Nonsense | c.89G>A | p.Trp30* | Muscular dystrophy, limb girdle |
| 2 | Nonsense | c.97C>T | p.Arg33* | Muscular dystrophy, limb girdle 2 |
| 3 | Missense | c.226G>T | p.Gly76Cys | Muscular dystrophy, limb girdle 2 |
| 4 | Nonsense | c.277G>T | p.Glu93T* | Muscular dystrophy, limb girdle 2 |
| 5 | Missense | c.391G>C | p.Ala131Pro | Muscular dystrophy, limb girdle |
| 6 | Nonsense | c.493C>T | p.Arg165T* | Muscular dystrophy, limb girdle |
| 7 | Missense | c.593G>C | p.Arg198Pro | Muscular dystrophy, limb girdle |
| 8 | Missense | c.631A>T | p.Asn211Tyr | Muscular dystrophy, limb girdle |
| 9 | Missense | c.784G>A | p.Glu262Lys | Muscular dystrophy, limb girdle |
| 10 | Small Deletion | c.382+1_382+2delGT | ------------------ | Muscular dystrophy, limb girdle |
| 11 | Small Deletion | c.657delC | ------------------ | Muscular dystrophy, limb girdle |
| 12 | Gross deletions | Exon 2-3 deleted | ------------------ | Muscular dystrophy |
| **13** | **Nonsense** | **c.289C>T** | **p.Arg97*** | **Muscular dystrophy, limb girdle 2** |
